# RAGE Is Essential for Subretinal Fibrosis in Laser-Induced Choroidal Neovascularization: Therapeutic Implications

**DOI:** 10.1167/iovs.66.6.30

**Published:** 2025-06-09

**Authors:** Parameswaran G. Sreekumar, Mi-Hyun Nam, Elise Hong, Ram Kannan, Ram H. Nagaraj

**Affiliations:** 1 Doheny Eye Institute, Pasadena, California, United States; 2 Sue Anschutz-Rodgers Eye Center and Department of Ophthalmology, School of Medicine, University of Colorado, Aurora, Colorado, United States

**Keywords:** age-related macular degeneration, epithelial–mesenchymal transition, subretinal fibrosis, retinal pigment epithelium, transforming growth factor-β2, RAGE

## Abstract

**Purpose:**

Subretinal fibrosis, a complication of neovascular age-related macular degeneration (nAMD), involves the epithelial–mesenchymal transition (EMT) of retinal pigment epithelium (RPE) cells as a contributing mechanism. The receptor for advanced glycation end products (RAGE) is a multiligand receptor implicated in fibrotic diseases, but its role in subretinal fibrosis has not been studied. This study investigated the role of RAGE in subretinal fibrosis.

**Methods:**

Subretinal fibrosis was induced in male RAGE^−/−^ and wild-type (WT) mice via laser photocoagulation, and fibrosis lesion volume was assessed on day 35 using optical coherence tomography and immunostaining. In vitro, EMT was induced in primary human RPE cells with transforming growth factor-beta 2 (TGF-β2). The role of RAGE in EMT was studied in cells pretreated with RAGE antagonists (FPS-ZM1 or azeliragon), followed by cotreatment with TGF-β2 for 48 hours. Signaling studies were conducted by pretreatment with FPS-ZM1 for 2 hours, cotreatment with TGF-β2 for 60 minutes, and subsequent immunoblot analysis.

**Results:**

In RAGE^−^^/^^−^ mice, subretinal fibrosis after laser-induced choroidal neovascularization was significantly reduced, with a smaller fibrosis volume, less inflammation, decreased activation of pSmad2, and reduced deposition of fibrotic markers (αSMA, collagen I) compared to WT mice. In vitro treatment with TGF-β2 in human RPE cells increased mitochondrial reactive oxygen species and upregulated EMT markers (αSMA, collagen I, and fibronectin), which were inhibited by cotreatment with FPS-ZM1 or azeliragon. FPS-ZM1 blocked TGF-β2–induced Smad2-dependent signaling and EMT without affecting the extracellular signal-regulated kinase (ERK) pathway.

**Conclusions:**

Our findings indicate that RAGE plays a role in RPE cell EMT in subretinal fibrosis and that RAGE antagonists attenuate this process, making RAGE a promising therapeutic target for subretinal fibrosis in nAMD.

Subretinal fibrosis, a hallmark endstage lesion in exudative or neovascular age-related macular degeneration (nAMD), results from abnormal repair processes.^1^ This fibrotic lesion typically follows progressive damage to the photoreceptors, retinal pigment epithelium (RPE), and choriocapillaris, leading to irreversible central vision loss.^2^^,^^3^ In nAMD patients, the prevalence of subretinal fibrosis increases significantly, rising from 13.0% to 37.8% within 1 year.^4^ Even with anti–vascular endothelial growth factor (VEGF) therapy, the risk of developing subretinal fibrosis remains substantial, with rates reported at 45% at 2 years^5^ and 41% at 10 years.^6^ These issues highlight the need for the development of new therapies to prevent subretinal fibrosis in nAMD.

Although the precise mechanisms underlying subretinal fibrosis are not fully understood, it is believed to result from a complex interaction of cellular and molecular factors. A key player in the development of subretinal fibrosis is the expression of extracellular matrix (ECM) by myofibroblasts.^7^^,^^8^ Myofibroblasts are not present in a healthy macula, suggesting that they arise from precursor cells in nAMD.^8^^,^^9^ RPE cells undergoing epithelial–mesenchymal transition (EMT) have been identified as the major source of myofibroblasts.^8^^–^^11^ In healthy eyes, RPE cells maintain a stable epithelial phenotype and are unable to proliferate due to effective cell–cell contact inhibition.^12^ However, when RPE cells lose their cell–cell contact and are exposed to growth factors, such as transforming growth factor-beta (TGF-β), they undergo a phenotypic transition, losing their epithelial characteristics. These changes are characterized by increased expression of mesenchymal markers, such as *N*-cadherin, vimentin, collagen I, and alpha smooth muscle actin (αSMA). As a result, RPE cells dissociate, acquire proliferative capacity, undergo EMT, deposit ECM proteins, and contribute to subretinal fibrosis in nAMD.^13^

The receptor for advanced glycation end products (RAGE), a member of the immunoglobulin receptor superfamily, is expressed in various cell types, including alveolar cells, macrophages, fibroblasts, and RPE cells.^14^^–^^17^ Although RAGE expression is typically low under normal physiological conditions, it is significantly upregulated during chronic inflammation and diabetes due to the accumulation of various RAGE ligands.^15^^,^^18^ RAGE is involved in several diseases, including diabetes, Alzheimer's disease, cardiovascular conditions, and secondary cataracts.^19^^–^^24^ Polymorphisms in the RAGE gene (rs1800624 and rs1800625) have been linked to an increased susceptibility to early and exudative age-related macular degeneration (AMD).^25^

In addition, RAGE plays a pivotal role in fibrosis in various organs, including the kidneys, heart, and liver. Studies have shown that, in the absence of RAGE, there is a reduction in endothelial-to-mesenchymal transition (EndMT) and autophagy-related protein expression. These properties are perceived to reduce cardiac fibrosis in mice subjected to transverse aortic coarctation.^26^ In liver fibrosis models, targeting the RAGE signaling pathway with small interfering RNA, anti-RAGE polyclonal antibodies, or recombinant sRAGE has been effective.^26^ RAGE has also been implicated in idiopathic pulmonary fibrosis, but the findings are controversial.^27^ Although a RAGE deficiency has been shown to protect against bleomycin-induced pulmonary fibrosis in mice,^28^^,^^29^ a previous report suggested that RAGE knockout worsens asbestos-induced pulmonary fibrosis.^30^ Additionally, deletion of RAGE had no effect on silica-induced pulmonary fibrosis.^31^ Similarly, some experimental models employing RAGE knockout mice have described a proinflammatory role for RAGE-mediated signaling in diet-induced murine nonalcoholic steatohepatitis.^32^^,^^33^ In contrast, others have shown conflicting results,^34^^,^^35^ suggesting that the RAGE signaling may vary by cell type and disease conditions.

Previously our research group has shown that the absence of RAGE retards the TGF-β–induced EMT in lens epithelial cells, suggesting that RAGE plays a role in fibrosis during posterior capsule opacification.^24^ In the present study, we tested the hypothesis that the absence of RAGE could reduce TGF-β2–induced EMT signaling in human RPE cells and decrease laser-induced subretinal fibrosis in RAGE^−/−^ mice. Our findings suggest that RAGE plays a pivotal role in the development of subretinal fibrosis by regulating the EMT of RPE cells through Smad signaling. In addition, RAGE antagonists significantly reduced EMT in RPE cells, suggesting that RAGE may be a potential target to prevent subretinal fibrosis in nAMD.

## Methods

### In Vivo Studies

The experimental protocols were approved by the Animal Research Committee at the University of California, Los Angeles (protocol ARC-2022-065) and were conducted in accordance with the National Institutes of Health guidelines and the ARVO Statement for the Use of Animals in Ophthalmic and Vision Research. RAGE^−/−^ mice (15–17 months old) were shipped from the University of Colorado (originally from Anne Marie Schmidt, NYU Langone Health) to the Doheny Eye Institute. Male C57BL6/J wild-type (WT) mice of the same age were purchased from The Jackson Laboratory (#000664; Bar Harbor, ME). All mice were housed in a standard experimental facility with a 12-hour light/dark cycle and were provided ad libitum access to food and water. Prior to experimental procedures, all animals were screened for the *rd8* mutation using previously published methods.^36^

### Laser-Induced Choroidal Neovascularization

Choroidal neovascularization (CNV) was induced as previously described.^37^^,^^38^ Mice were anesthetized, and their pupils were dilated with atropine (1% w/v) and phenylephrine hydrochloride (2.5% w/v) (Covetrus, Portland, ME, USA). The fundus was visualized using an imaging camera, and laser photocoagulation was performed using an image-guided laser system (MICRON IV; Phoenix Research Laboratories, Pleasanton, CA, USA). Four laser burns, equidistant from the optic nerve, were applied to each eye using a green Argon laser pulse (532-nm wavelength, 50-µm spot diameter, 70-ms exposure duration, 240-mW power). Spots exhibiting subretinal hemorrhage were excluded from the analysis. Successful rupture of Bruch's membrane was confirmed using a digital image-guided spectral-domain optical coherence tomography (SD-OCT) system (MICRON IV). Following laser treatment, the eyes were rinsed with sterile saline and treated with erythromycin ointment (Covetrus). Mice were then placed on a preheated warming pad at 37°C until fully recovered.

### Spectral-Domain Optical Coherence Tomography

On day 35 post-laser, mouse pupils were dilated with atropine and phenylephrine hydrochloride drops after anesthesia induction with a xylazine–ketamine mixture. SD-OCT, guided by brightfield live fundus imaging, was performed using the MICRON IV image-guided OCT system following the manufacturer's instructions. The vendor's image acquisition software was used to generate OCT scans, and lesion areas were quantified using Image J (National Institutes of Health, Bethesda, MD, USA).

### CNV and Subretinal Fibrosis Volume Measurement

The volumes of CNV and subretinal fibrous tissue were measured in RPE/choroidal flatmounts at day 35 post-laser photocoagulation, following the procedures described in our previous publication.^38^ Briefly, mouse eye cups were fixed in 4% paraformaldehyde and permeabilized with 1% Triton X-100 for 2 hours. After removal of the anterior segment and neural retina, the flatmounts were stained with the CNV marker fluorescein-labeled isolectin-B4 (1:100 dilution, #FL1201; Vector Laboratories, Newark, CA, USA) and the fibrosis marker collagen type I (1:100 dilution, #72026; Cell Signaling Technology, Danvers, MA, USA), and were incubated overnight at 4°C. Following incubation with the secondary antibody against collagen I and nuclear staining with Hoechst, samples were mounted with VECTASHIELD medium (Vector Laboratories) and examined using a confocal microscope (LSM 710; Carl Zeiss Microscopy, Oberkochen, Germany). Fluorescence volume measurements were obtained by generating z-stacks of optical slices within the lesions. Image stacks were rendered in three dimensions using Velocity imaging software (Improvision, Waltham, MA, USA) and processed to digitally extract the fluorescent lesion volume. CNV volume was quantified in cubic micrometers.^38^

### Immunostaining of αSMA, Collagen I, CD206, pSmad2, and RAGE in Fibrotic Lesions

Eye cups were cryopreserved and serially sectioned, and 8-µm sections were fixed in 4% paraformaldehyde for 20 minutes at room temperature. The sections were then permeabilized with 0.5% Triton X-100 for 5 minutes. After permeabilization, the sections were blocked with an animal-free blocking reagent (#SP-5035-100; Vector Laboratories) for 30 minutes. The sections were then incubated overnight at 4°C with primary antibodies against αSMA (1:100 dilution; Sigma-Aldrich, St. Louis, MO, USA), collagen I (1:100 dilution; Cell Signaling Technology), CD206 (1:1000 dilution; Santa Cruz Biotechnology, Dallas, TX, USA), pSmad2 (1:100 dilution, #3108; Cell Signaling Technology), and RAGE (1:75 dilution, #sc-365154; Santa Cruz Biotechnology) in the blocking buffer. Blocking buffer without the primary antibody was used as the negative control. Following incubation, the sections were washed with PBS and then incubated with the appropriate secondary antibodies (Vector Laboratories) for 1 hour at room temperature. The specimens were mounted in a mounting medium and examined using an LSM 710 confocal microscope. Fluorescence intensity levels of αSMA, collagen I, pSmad2, and RAGE were quantified using ImageJ software.^39^

### Primary Human RPE Cell Culture

All procedures adhered to the tenets of the Declaration of Helsinki for research involving human subjects. The RPE cells were isolated from human fetal eyes received from Advanced Bioscience Resources (Alameda, CA, USA) and cultured as previously described.^40^^–^^42^ Confluent cell cultures between passages 2 and 4 were used in all experiments. In brief, human RPE (hRPE) cells were grown in Dulbecco's Modified Eagle Medium (DMEM, #15-013-CV; Corning Inc., Corning, NY, USA) with 10% fetal bovine serum (FBS, #4800-500HI; Laguna Scientific, Laguna Niguel, CA, USA) containing l-glutamine (25030149; Thermo Fisher Scientific, Waltham, MA, USA) and antibiotic–antimycotic (#15240062; Thermo Fisher Scientific).

Subconfluent hRPE cells (60%–70% confluence in 0.1% fetal bovine serum [FBS]) were used in experiments with recombinant human TGF-β2 (#GF113; Sigma-Aldrich) and FPS-ZM1 (#553030; MilliporeSigma, Burlington, MA, USA). To investigate the effect of the RAGE antagonist FPS-ZM1 on RPE EMT, hRPE cells were pretreated with 20-µM FPS-ZM1 for 2 hours, followed by cotreatment with TGF-β2 (10 ng/mL) and FPS-ZM1 for 48 hours. For signaling pathway analysis, cells were pretreated with 20-µM FPS-ZM1 for 2 hours, then cotreated with TGF-β2 (10 ng/mL) and FPS-ZM1 for 60 minutes. At the end of each experiment, samples were collected for immunoblot analysis. To further confirm the findings from the first RAGE antagonist, we also used another RAGE antagonist, azeliragon (TTP488, #HY-50682; MedChemExpress, Monmouth Junction, NJ, USA), and followed the same treatment protocol described above.

### Cell Viability Analysis

hRPE cells (25,000 per well) were cultured in 96-well plates and treated with varying concentrations of FPS-ZM1 or azeliragon in culture medium containing 0.1% FBS for 48 hours. After treatment, Alamar Blue reagent (#DAL1025; Thermo Fisher Scientific) was added to each well (final dilution, 1:10) and incubated for 3 hours. Fluorescence was measured using 560-nm excitation and 590-nm emission on a SpectraMax iD5 Multi-Mode Microplate Reader (Molecular Devices, San Jose, CA, USA). Data are presented as the percentage change in cell viability.

### Detection of Mitochondrial Superoxide Using MitoSOX

Mitochondrial superoxide production was assessed using MitoSOX (#M36008; Thermo Fisher Scientific). hRPE cells were seeded onto four-well chamber slides and pretreated with FPS-ZM1 (20 µM) or azeliragon (1 µM) for 2 hours. Following pretreatment, cells were co-incubated with TGF-β2 for 24 hours. Prior to the termination of the treatment, cells were exposed to MitoSOX (5 µM) for 15 minutes at 37°C. Subsequently, cells were rinsed with PBS, fixed with 4% paraformaldehyde for 10 minutes, and analyzed using a LSM 710 laser scanning confocal microscope. The mean fluorescence intensity of MitoSOX after treatment was quantified using ZEISS ZEN microscopy software. The data are expressed as the mean fluorescence intensity.

### Immunoblot Analysis

At the end of the experiment, hRPE cells were rinsed twice with PBS, and protein extraction was performed using the Mammalian Protein Extraction Reagent (M-PER, #78501; Thermo Fisher Scientific), supplemented with a protease and phosphatase inhibitor cocktail (1:100 dilution, #78447; Thermo Fisher Scientific). After gentle shaking for 5 minutes at room temperature, the lysates were centrifuged at 14,000*g* for 10 minutes at 4°C, and the supernatants were collected. Protein concentration was measured using the Bio-Rad Protein Assay (Bio-Rad Laboratories, Hercules, CA, USA), following the manufacturer's instructions.

Equal amounts of protein (20 µg) were loaded onto Mini-PROTEAN TGX Precast Protein Gels (Bio-Rad) and transferred to polyvinylidene fluoride (PVDF) membranes (#IPVH00010; MilliporeSigma). The membranes were then incubated overnight at 4°C with primary antibodies against αSMA, collagen I, fibronectin, or GAPDH in 5% nonfat dry milk (#170-6404; Bio-Rad). After incubation with the corresponding secondary antibodies (Vector Laboratories), protein bands were detected using the SuperSignal West Pico PLUS Chemiluminescence substrate (#34580; Thermo Fisher Scientific). The antibodies used for western blotting are listed in the Table.

**Table.** Antibodies Used in This Study

Antibody	Source	Dilution	Supplier
αSMA	Mouse	1:2000	MilliporeSigma (#A2547)
Fibronectin	Mouse	1:1000	Santa Cruz Biotechnology (#sc-8422)
Collagen I	Mouse	1:500	Santa Cruz Biotechnology (#sc-293182)
GAPDH	Mouse	1:5000	MilliporeSigma (MAB374)
p-Smad2	Rabbit	1:1000	Cell Signaling Technology (#3108S)
Smad2	Rabbit	1:1000	Cell Signaling Technology (#5339S)
p-ERK	Rabbit	1:1000	Cell Signaling Technology (#4370S)
CD206	Mouse	1:100	Santa Cruz Biotechnology (#sc-58986)
ERK	Rabbit	1:1000	Cell Signaling Technology (#4695S)
β-actin	Rabbit	1:5000	Cell Signaling Technology (#5125S)
Anti-mouse IgG	Goat	1:5000	Cell Signaling Technology (#7076S)
Anti-rabbit IgG	Goat	1:5000	Cell Signaling Technology (#7074S)

### Data Analysis

All data are expressed as mean ± SEM. Data were analyzed using one-way ANOVA followed by Tukey’s post hoc test using Prism 5 (GraphPad Software, Boston, MA, USA). *P* < 0.05 was considered statistically significant.

## Results

### Attenuation of Subretinal Fibrosis in RAGE^−/−^ Mice

**Figure 1.**
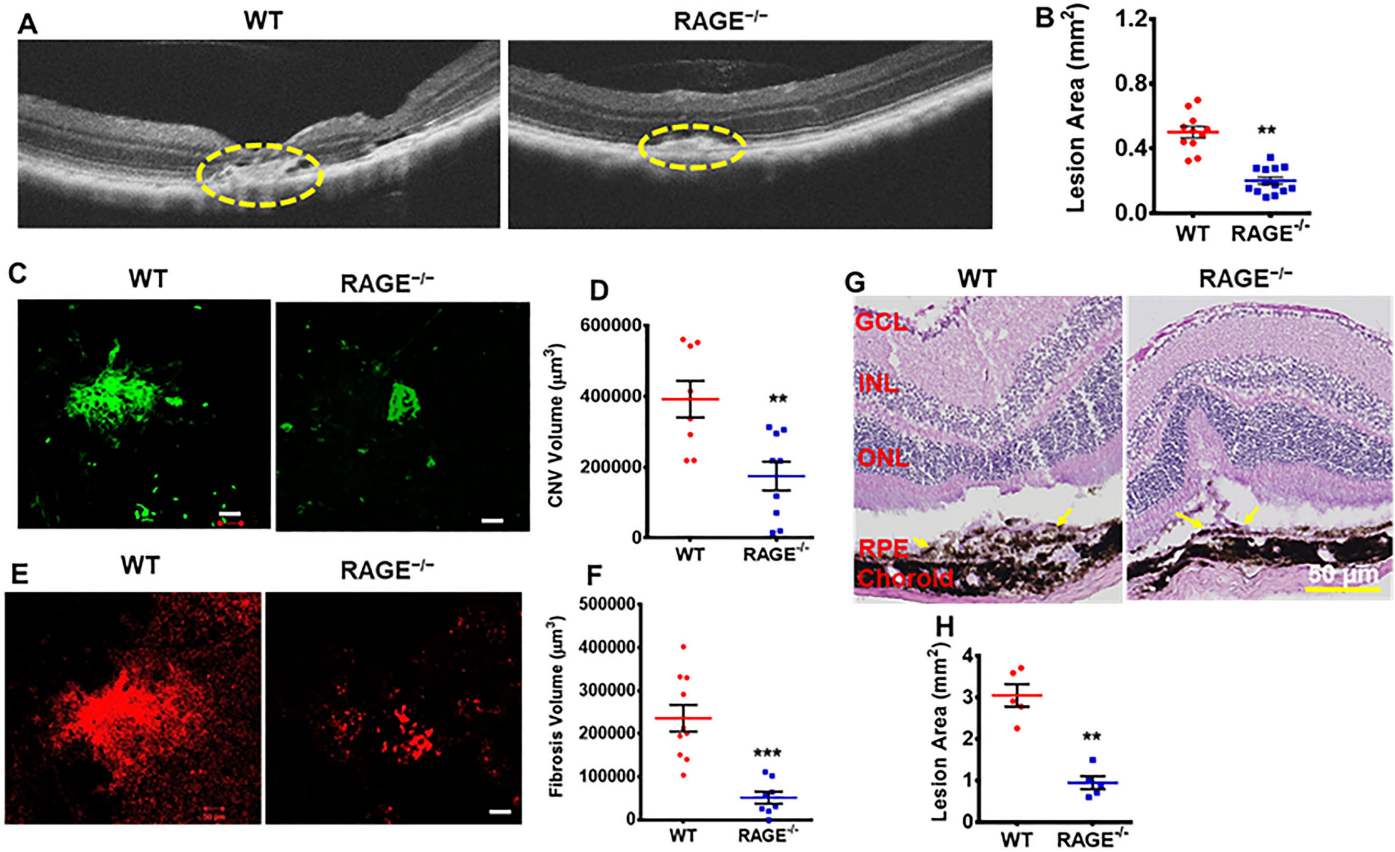
Fibrosis volume is decreased in RAGE^−/−^ mice following laser photocoagulation. (**A**) Representative OCT images from lasered WT and RAGE^−/−^ mice on day 35. *Yellow circles* indicate lesions. (**B**) Quantification of lesion area using Image J (*n* = 11–13; mean ± SEM). **P < 0.01 (unpaired *t*-test). (**C, D**) Representative confocal images of RPE/choroid flatmounts stained for isolectin B4 (*green*) (**C**) and CNV volume quantification (**D**) from WT and RAGE^−/−^ mice, 35 days post-laser. *Scale bar*: 50 µm. (**E, F**) Fibrosis marker (collagen I, *red*) (**E**) and mean volume of fibrosis at 35 days post-laser (**F**) (*n* = 8–10 lesions from 4–5 mice per group; mean ± SEM) ** *P* < 0.01, *** *P* < 0.001 (unpaired *t*-test). (**G**) Histology of retinal sections showing lesions in WT and RAGE^−/−^ mice on day 35 post-laser. *Yellow arrows* indicate lesions. *Scale bar*: 50 µm. (**H**) Quantification of lesion areas using Image J (*n* = 5 mice per group; mean ± SEM). **P < 0.1 (unpaired *t*-test).

The RAGE^−/−^ mice carried a reporter green fluorescent protein, the expression of which and its inherent fluorescence hindered our ability to capture better fundus images. However, despite this limitation, our CNV technique resulted in the breakage of Bruch's membrane, as evident in Supplementary Figure S1. OCT images were captured on day 35 post-laser. Analysis of lesion size from two-dimensional images revealed a significant difference between WT and RAGE^−/−^ mice, with smaller lesions in the RAGE^−/−^ mice (Figs. 1A, 1B). We then isolated RPE/choroid flatmounts, stained for CNV using isolectin B4 (green) and for fibrosis with collagen I (red), and quantified CNV and fibrosis volume by creating z-stacks of optical slices from the lesions, visualizing them in three dimensions with velocity imaging software. Our data revealed a significant reduction in CNV volume in RAGE^−/−^ mice compared to WT controls (Figs. 1C, 1D). In parallel, we assessed the subretinal fibrosis by evaluating collagen I (red), a marker of fibrosis. The subretinal fibrosis volume was significantly increased in the WT mice following laser treatment. In contrast, RAGE^−/−^ mice exhibited a marked reduction in the subretinal fibrosis volume (Figs. 1E, 1F). To validate these findings, we conducted histological analysis and quantified lesion areas. Consistent with the OCT data, histological assessment revealed larger lesions in WT mice compared to RAGE^−/−^ mice (Figs. 1G, 1H). These results suggest a critical role for RAGE in subretinal fibrosis, with its absence mitigating fibrosis in laser-induced CNV.

### Elevated RAGE Expression in the Fibrotic Retina of Day 35 Post-Laser Mice

**Figure 2.**
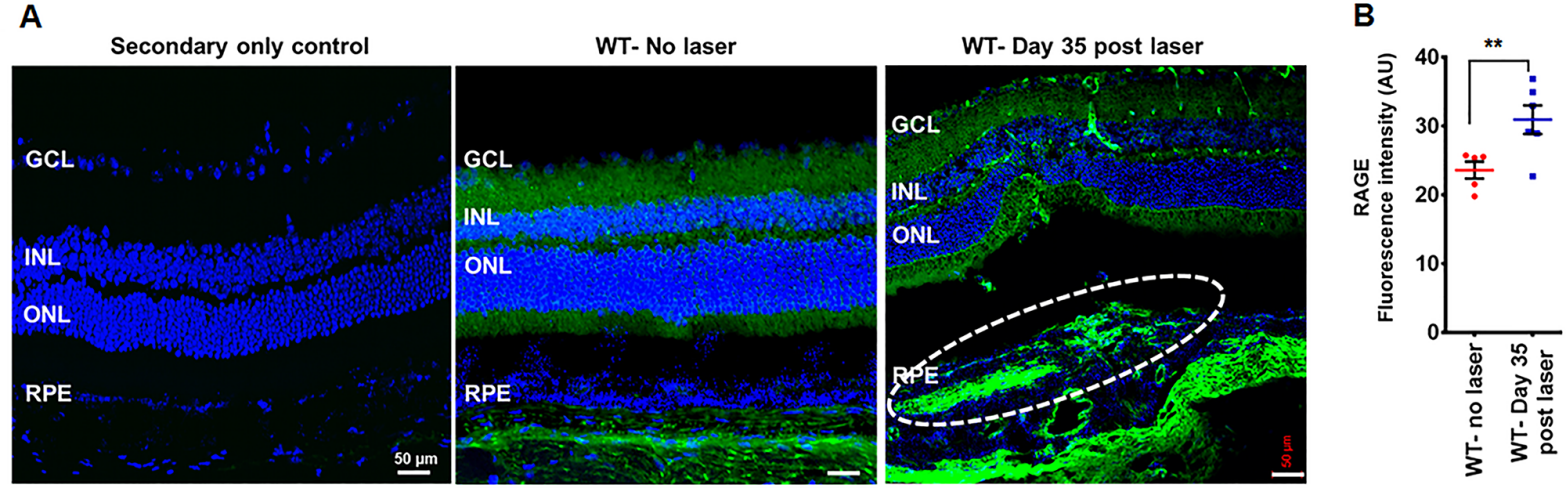
RAGE expression was higher in day 35 post-laser mice. (**A**) Representative confocal images of retinal cryosections from WT control (unlasered) and WT lasered (day 35 post-laser) mice showing RAGE staining (*green*). Cell nuclei were counterstained with 4′,6-diamidino-2-phenylindole (DAPI, *blue*). Negative control staining with secondary antibody only is shown. (**B**) Quantification of RAGE expression was performed using ImageJ (*n* = 5 or 6 retinal sections per group; mean ± SEM). *Scale bar*: 50 µm. **P < 0.01 (unpaired *t*-test).

Given that RAGE expression increases during CNV^43^ and fibrosis is enhanced in WT mice, we sought to investigate RAGE expression in the retina of day 35 post-laser WT mice. RAGE expression was notably elevated in the fibrotic retina of day 35 post-laser WT mice. In unlasered WT mice, as reported previously,^43^ RAGE was present at low levels in the inner plexiform layer, outer plexiform layer, inner segments of photoreceptors, and the RPE monolayer (Fig. 2A). In contrast, a significant increase in RAGE immunoreactivity was observed on day 35 post-laser treatment (Fig. 2B). The strongest RAGE expression was localized to the fibrotic lesions (within the dotted white lines), highlighting a potential link between RAGE and subretinal fibrosis. Additionally, the RAGE staining pattern within the neural retina was selectively enhanced in specific cell types (Fig. 2A), suggesting that RAGE may play a role in the pathogenesis of subretinal fibrosis. We did not observe morphological differences between 26-week-old WT and RAGE^−/−^ retinas (Supplementary Fig. S2).

### αSMA and Collagen I Expression in CNV/Subretinal Fibrosis Lesions

**Figure 3.**
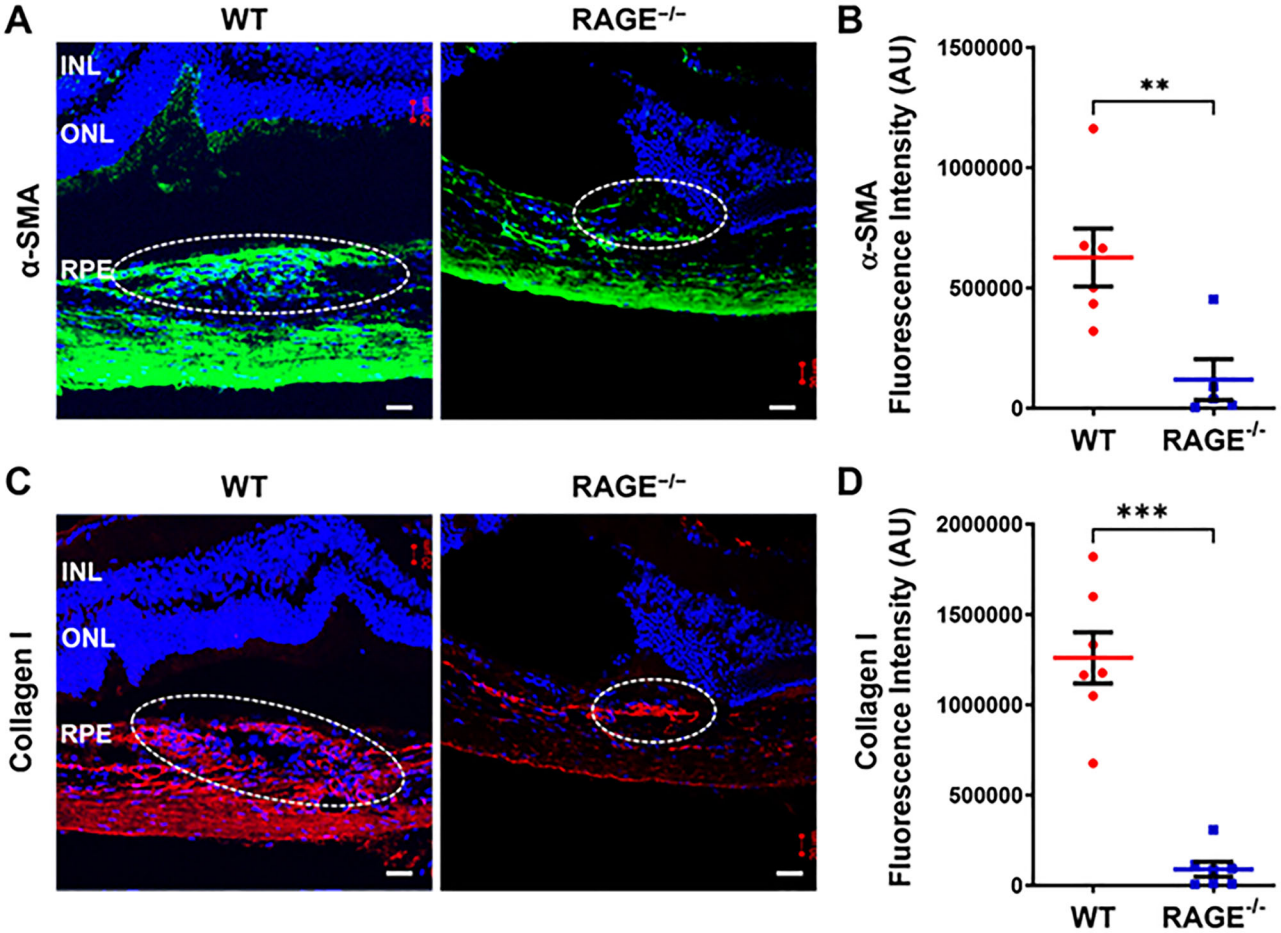
RAGE^−/−^ mice showed reduced fibrosis. (**A**–**D**) Representative confocal images of retinal cryosections from WT and RAGE^−/−^ mice showed subretinal fibrotic lesions stained for αSMA (*green*) (**A**) and collagen I (*red*) (**C**). Cell nuclei were counterstained with DAPI (*blue*). Quantification of αSMA (**B**) and collagen I (**D**) content was performed using Image J (*n* = 5–7 retinal sections, corresponding to 5–7 mice per group; mean ± SEM). *Scale bar*: 20 µm. **P < 0.01, ***P < 0.01 (unpaired *t*-test).

OCT and hematoxylin and eosin (H&E) staining of the retinal–choroidal complexes clearly showed laser-induced lesions with the rupture of Bruch's membrane (Figs. 1A, 1G). Immunostaining revealed a significant reduction in αSMA, a marker for activated myofibroblasts, in the RAGE^−/−^ mice compared to the corresponding WT controls in retinal sections at day 35 post-laser (Figs. 3A, 3B). Additionally, collagen I, an ECM protein associated with scar formation, was significantly decreased in the RAGE^−/−^ mice compared to the WT mice at day 35 post-laser (Figs. 3C, 3D). These findings suggested that the absence of RAGE leads to reduced scar formation and inhibits myofibroblast activation in response to laser-induced retinal injury.

### Reduced Macrophage Infiltration and Smad2 Activation in RAGE^−/−^ Mice

**Figure 4.**
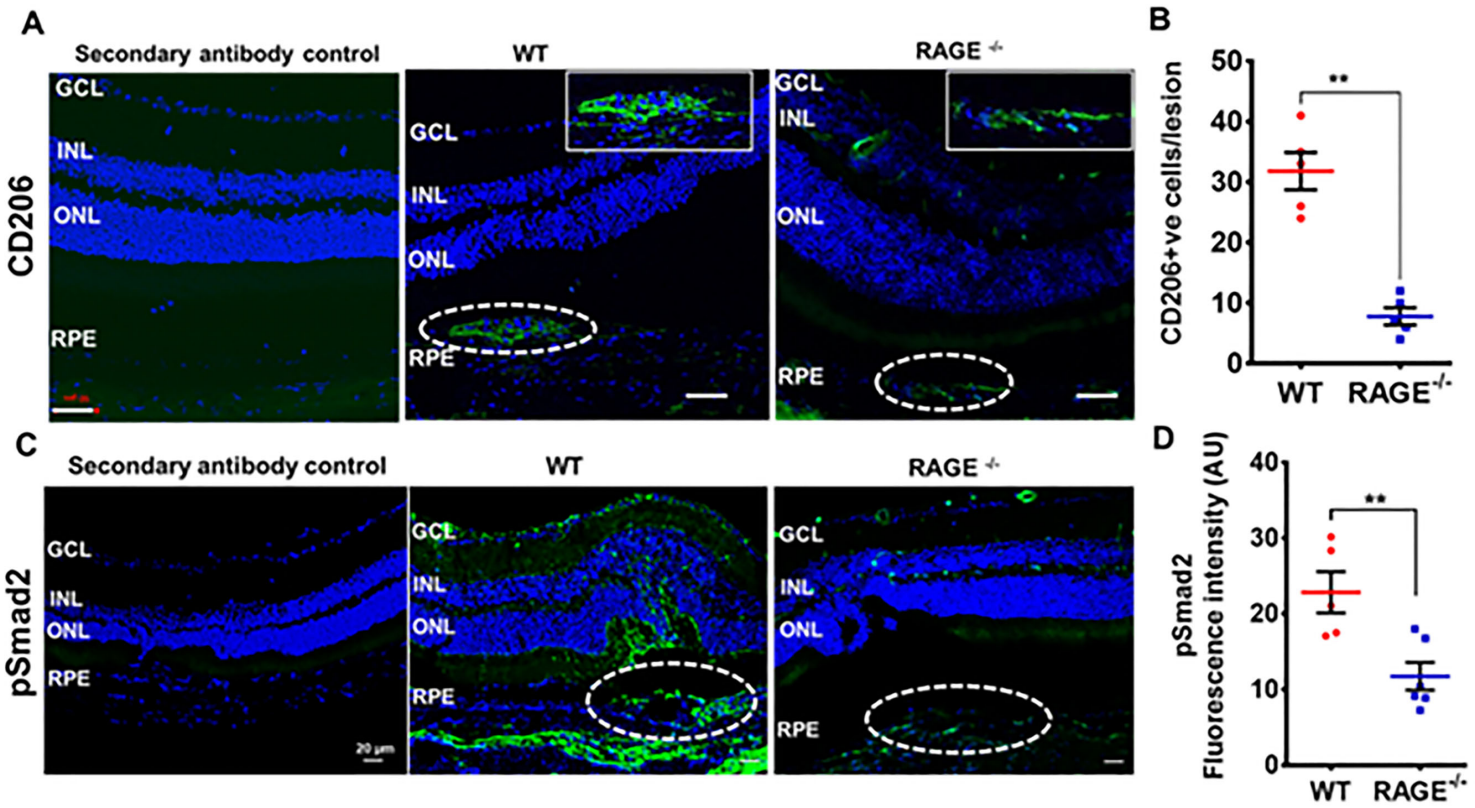
RAGE deficiency prevented macrophage infiltration and inhibited Smad2 activation and subretinal fibrosis. (**A**) Representative confocal images of retinal cryosections from WT and RAGE^−/−^ mice show subretinal fibrotic lesions stained for M2 macrophages (CD206, *green*), with cell nuclei counterstained with DAPI (*blue*). *Scale bar*: 50 µm. (**B**) Quantification of the number of CD206^+^ cells in subretinal fibrotic lesions (within the *dotted white lines*) of WT and RAGE^−/−^ mice. Only cells positive for both the nuclear stain (*blue*) and CD206 (*green*) within the lesion area were counted*.* The *inset* shows a magnified view of the lesion area, outlined by *white dotted lines*. Data are presented as mean ± SEM (*n* = 4 or 5 sections per group from four or five mice per group). **P < 0.1 (Student’s *t*-test). (**C**) Confocal images showing pSmad2 staining in retinal sections from lasered WT and RAGE⁻^/^⁻ mice. Sections incubated with secondary antibody only (without the primary antibody) served as negative controls. (**D**) Quantification of pSmad2 immunoreactivity was performed using ImageJ. Data are presented as mean ± SEM (*n* = 4–6 sections per group from four or five mice per group). **P < 0.1 (Student’s *t*-test).

Macrophages are key angiogenic effector cells, with M1 and M2 phenotypes observed in the eye. Initially, M1 macrophages are inflammatory but later shift to the anti-inflammatory, pro-angiogenic, profibrotic M2 phenotype, which predominates in advanced CNV. Therefore, we investigated M2 macrophage expression in retinal sections at day 35 post-laser treatment. CD206 was used as an M2 macrophage marker. The results showed that CD206^+^ cells were concentrated in and around the fibrotic lesions (Fig. 4A). Compared to WT mice, significantly fewer CD206^+^ cells were found in RAGE^−/−^ mice (*P* < 0.001) (Fig. 4B).

Given that the TGF-β/Smad2 signaling axis plays a central role in the development and progression of fibrosis,^1^ we examined the expression levels of phosphorylated Smad2 (pSmad2) in retinal sections from WT and RAGE^−/−^ mice at day 35 post-laser treatment. pSmad2 expression was significantly increased (*P* < 0.001) in the lasered WT retinas, with particularly strong immunoreactivity observed in fibrotic lesion areas (Figs. 4C, 4D). In contrast, pSmad2 phosphorylation was markedly reduced in the retinas of lasered RAGE^−/−^ mice, suggesting that RAGE may contribute to Smad2 activation in this fibrotic response.

### RAGE Antagonists (FPS-ZM1 and Azeliragon) Inhibited the TGF-β2–Induced EMT in hRPE Cells

To investigate the role of RAGE in RPE-EMT, we used TGF-β2 to induce EMT in hRPE cells. First, to assess the effects of a RAGE inhibitor on hRPE cell viability, cells were treated with FPS-ZM1 at concentrations ranging from 5 µM to 25 µM. The results demonstrated that FPS-ZM1 had no significant effect on cell viability at any of the concentrations tested (Supplementary Fig. S3A). The effects of FPS-ZM1 (20 µM) on RPE EMT were evaluated. Cells were pretreated with FPS-ZM1 for 2 hours, followed by co-incubation with TGF-β2 for 48 hours.

**Figure 5.**
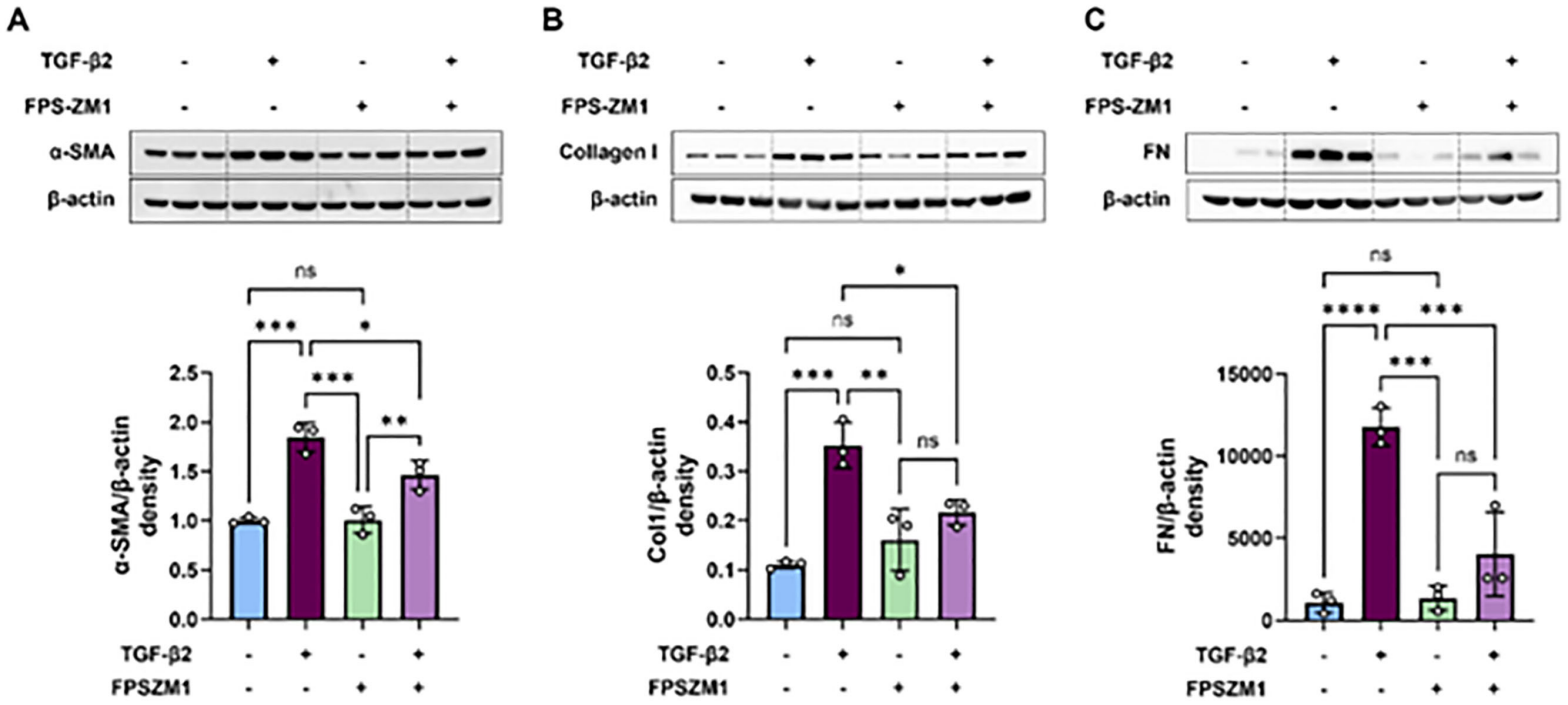
RAGE antagonist FPS-ZM1 treatment inhibited TGF-β2–induced EMT in hRPE cells. hRPE cells were pretreated with either 10 or 20 µM of FPS-ZM1 for 2 hours in DMEM containing 0.1% FBS, followed by cotreatment with TGF-β2 (10 ng/mL) and FPS-ZM1 for 48 hours. (**A**–**C**) Cell lysate proteins were subjected to western blot analysis for αSMA (**A**), collagen I (**B**), and fibronectin (**C**). The quantitative densitometry data from three to four independent experiments are presented as bar graphs. TGF-β2 treatment significantly increased the expression of EMT markers, whereas cotreatment with FPS-ZM1 markedly inhibited their expression. Data are shown as mean ± SEM (*n* = 3). *P < 0.05, **P < 0.01, ***P < 0.001, ****P < 0.0001 (one-way ANOVA, Tukey's multiple comparison test); ns, not significant. Col I, collagen I; FN, fibronectin.

Western blot analysis revealed a significant upregulation of EMT markers (αSMA, fibronectin, and collagen I) by TGF-β2, which was substantially inhibited by FPS-ZM1 treatment (Figs. 5B, 5C). Additionally, immunofluorescence analysis of αSMA in hRPE cells treated with TGF-β2 and FPS-ZM1 (5 µM) showed a similar trend (Supplementary Fig. S4). FPS-ZM1 alone did not notably affect the expression of these markers (Figs. 5B, 5C).

**Figure 6.**
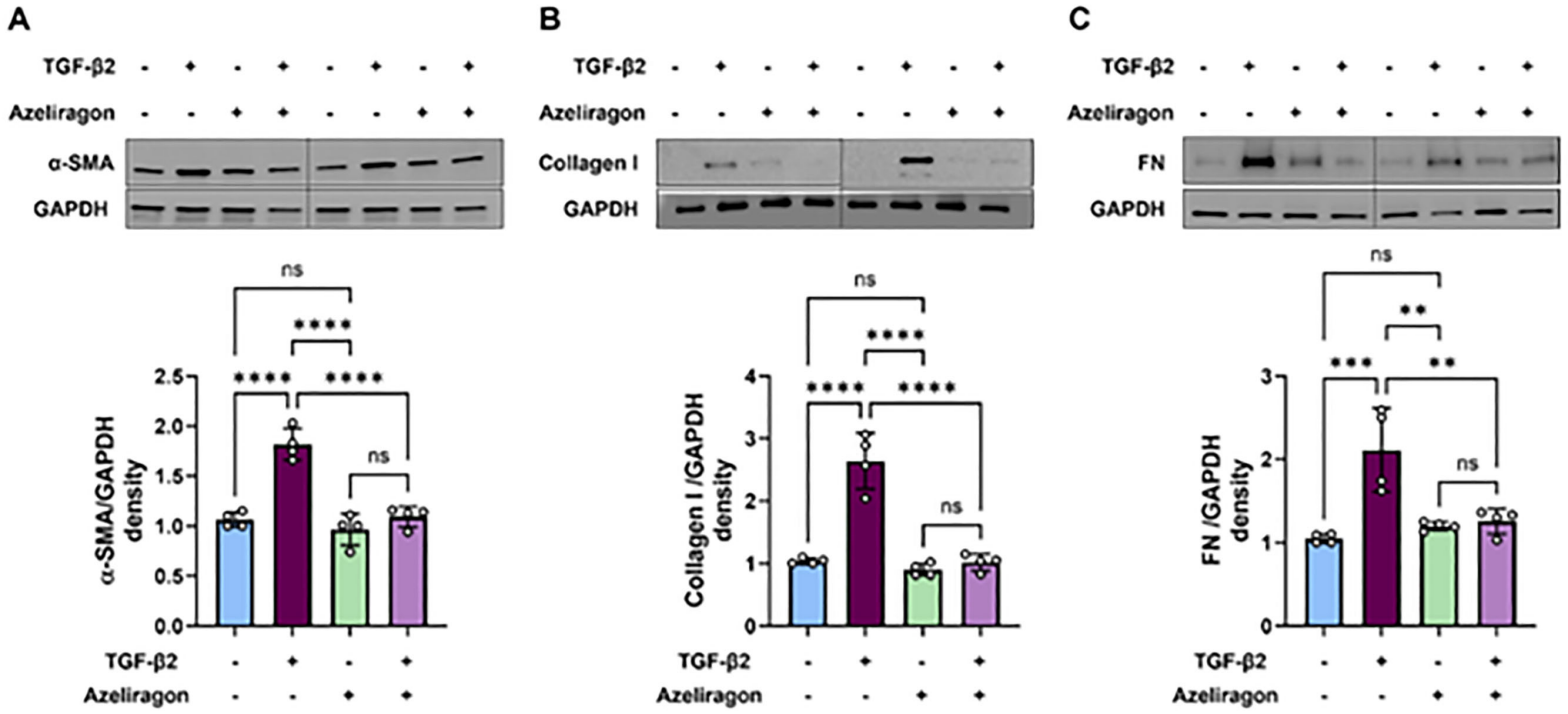
RAGE antagonist azeliragon blocked TGF-β2–induced EMT in hRPE cells. hRPE cells were pretreated with 1 µM azeliragon for 2 hours in DMEM containing 0.1% FBS, followed by cotreatment with TGF-β2 (10 ng/mL) and azeliragon for 48 hours. (**A**–**C**) At the end of the experiment, proteins were isolated and analyzed using western blot for the EMT markers αSMA (**A**), collagen I (**B**), and fibronectin (**C**). The quantitative densitometry data from three to four independent experiments are presented as bar graphs. TGF-β2 treatment significantly increased the expression of EMT markers, whereas treatment with azeliragon notably inhibited their expression. The quantitative densitometry data from four independent experiments are presented as bar graphs. The data are shown as mean ± SEM (*n* = 4). **P < 0.01, ***P < 0.001, ****P < 0.0001 (one-way ANOVA, Tukey's multiple comparison test); ns, not significant. Col I, collagen I; FN, fibronectin; Aze, azeliragon.

To ensure the specificity of the effects of the above RAGE antagonist, we tested a second RAGE antagonist, azeliragon (TTP488). hRPE cells were treated with azeliragon at concentrations ranging from 0.5 µM to 3 µM for 48 hours. The results showed no significant effect on cell viability (Supplementary Fig. S3B). Based on the cell viability data, we selected a concentration of azeliragon of 1 µM for all EMT studies. hRPE cells were pretreated with azeliragon, for 2 hours, followed by TGF-β2 for 48 hours. Western blot analysis revealed a significant downregulation of all EMT markers with azeliragon treatment (Figs. 6A–6C), further supporting the role of RAGE in the TGF-β2–induced RPE EMT.

### Exposure of hRPE to TGF-β2 Enhanced Mitochondrial Superoxide Production, Which Was Inhibited by RAGE Antagonists

**Figure 7.**
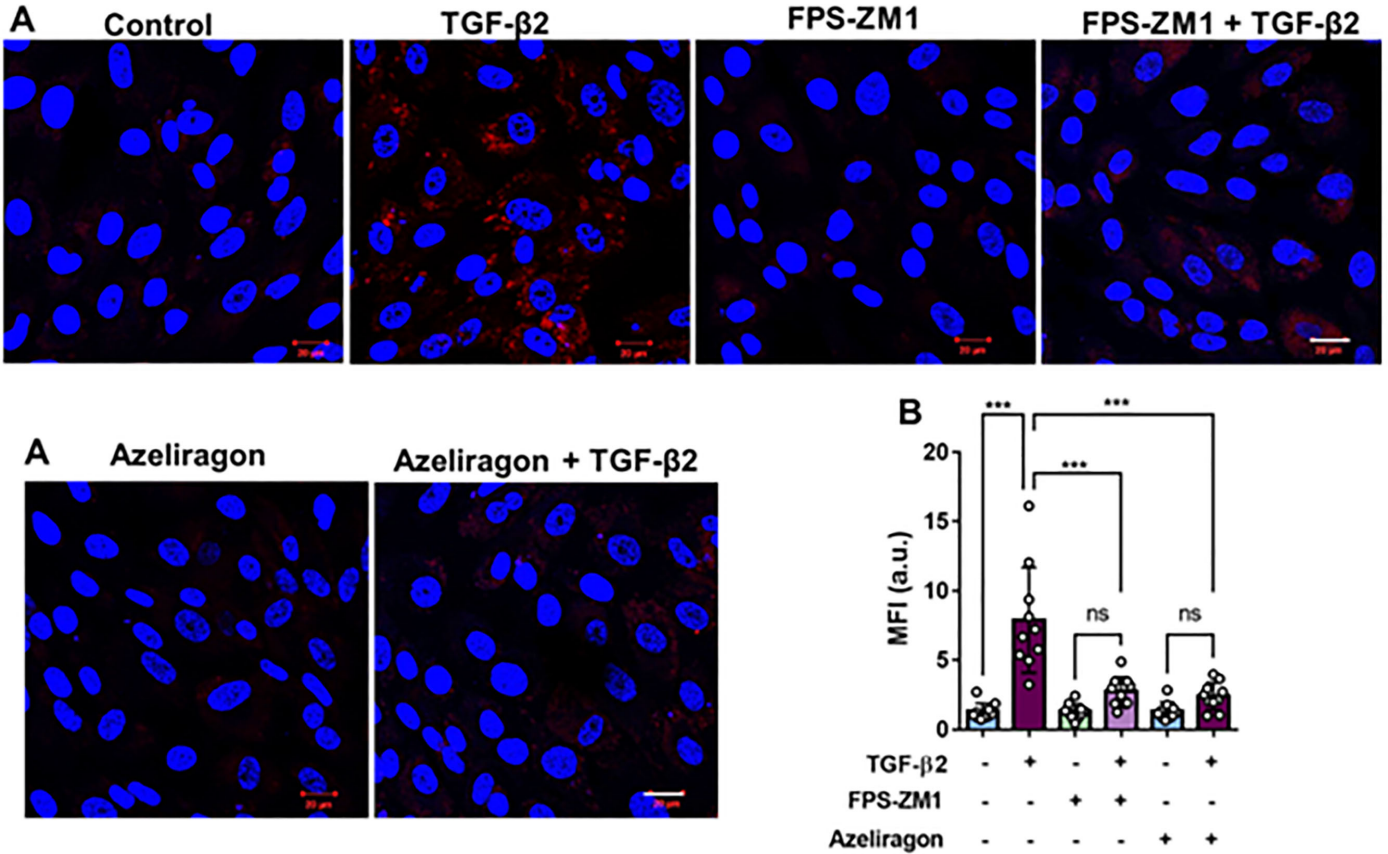
RAGE inhibition blocks TGF-β2–induced mitochondrial superoxide production in hRPE cells. (**A**) hRPE cells were pretreated with RAGE antagonists FPS-ZM1 (20 µM) or azeliragon (1 µM) for 2 hours, followed by cotreatment with TGF-β2 and the RAGE antagonist in DMEM containing 0.1% FBS for 24 hours. Cells were then stained with MitoSOX to detect mitochondrial superoxide production (MitoSOX, *red*; DAPI, *blue*). *Scale bar*: 20 µm. (**B**) Fluorescence intensities from the experiment in **A** were quantified using ZEISS ZEN software. TGF-β2–treated cells exhibited significantly increased mitochondrial superoxide production, whereas RAGE antagonist pretreated cells showed significantly reduced superoxide production (*n* = 10; mean ± SEM). ***P < 0.001 (ANOVA, Tukey's post hoc test); ns, not significant.

To determine whether TGF-β2 induces oxidative stress and enhances mitochondrial superoxide, hRPE cells were stained with MitoSOX Red, a dye that detects reactive oxygen species (ROS) generated within the mitochondria. Mitochondrial ROS levels were imaged using confocal microscopy. As shown in Figures 7A and 7B, the TGF-β2–treated group displayed a significant increase in mitochondrial ROS compared to the control group. Treatment with RAGE antagonists alone did not significantly alter mitochondrial ROS generation. However, TGF-β2–induced mitochondrial ROS production was significantly inhibited in the RAGE antagonist-treated groups, suggesting that RAGE is involved in regulating mitochondrial function (Figs. 7A, 7B).

### FPS-ZM1 Inhibited TGF-β2–Induced EMT in hRPE Cells by Suppressing the Smad2 Signaling Pathway

**Figure 8.**
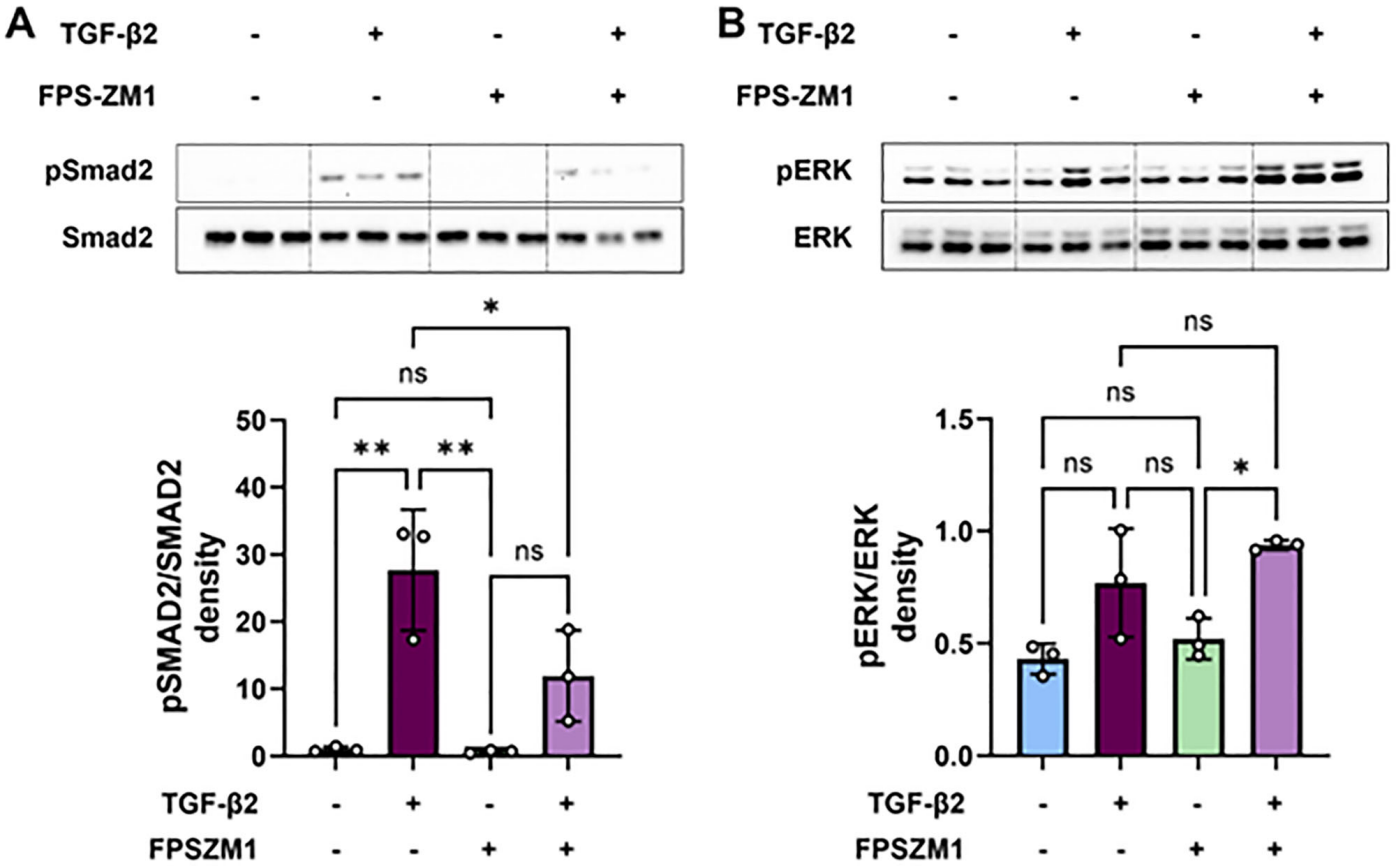
The RAGE antagonist FPS-ZM1 attenuated TGF-β2–mediated Smad2 phosphorylation in hRPE cells. hRPE cells were pretreated with 20 µM of FPS-ZM1 for 2 hours in DMEM containing 0.1% FBS, followed by cotreatment with TGF-β2 (10 ng/mL) and FPS-ZM1 for 60 minutes. (**A**, **B**) Cell lysate proteins were analyzed by western blot to assess the regulation of canonical signaling (pSmad2, total Smad2) (**A**) and non-canonical signaling (pERK and total ERK) (**B**). Quantitative densitometric data, derived from three independent experiments, are presented as bar graphs. TGF-β2 treatment significantly activated Smad2, which was inhibited by the RAGE antagonist. However, no significant activation of ERK was observed. Densitometric analysis of the blots, normalized to total Smad2, is shown in the bar graphs (*n* = 3; mean ± SEM). *P < 0.05, **P < 0.01 (one-way ANOVA, Tukey's multiple comparison test); ns, not significant.

To explore the molecular mechanisms through which RAGE is involved in EMT, we examined signaling pathways associated with EMT, including the canonical TGF-β/Smad signaling and non-canonical extracellular signal-regulated kinase (ERK) signaling. We observed Smad2 phosphorylation in hRPE cells treated with TGF-β2 for 60 minutes using western blot analysis. Phosphorylated Smad2 was significantly elevated (*P* < 0.05) in hRPE cells exposed to TGF-β2 for 60 minutes compared to untreated cells, and this elevation was significantly inhibited by the RAGE antagonist FPS-ZM1 (Fig. 8A). Treatment with FPS-ZM1 alone did not significantly affect the pSmad2 levels. Treatment with TGF-β2, FPS-ZM1 alone, or their combination did not affect ERK phosphorylation.

## Discussion

This study investigated the role of RAGE in the development of subretinal fibrosis and provides strong evidence for its role in the pathogenesis of subretinal fibrosis. In a laser-induced choroidal neovascularization model, subretinal fibrosis was significantly attenuated in RAGE^−/−^ mice, as evidenced by reduced inflammation, decreased Smad activation and lower extracellular matrix accumulation, highlighting the importance of RAGE in subretinal fibrosis. Conversely, in WT mice, RAGE expression was significantly upregulated on day 35 post-laser treatment, with increased localization of RAGE in the lesion area and ganglion cell layer. Additionally, we demonstrated that blocking of RAGE prevented TGF-β2–induced mitochondrial superoxide production and EMT in RPE cells by reducing activation of the Smad2 signaling pathway. Together, these findings suggest, for the first time, that RAGE plays a critical role in subretinal fibrosis and may represent a promising therapeutic target for mitigating subretinal fibrosis.

The involvement of RAGE in both homeostatic and pathological processes is well established, with prior studies identifying its role in fibrosis across various animal models.^26^^,^^44^^–^^47^ Specifically, RAGE^−/−^ mice exhibited reduced CNV lesion sizes and fewer infiltrating cells, suggesting that RAGE contributes to inflammatory processes during CNV progression.^43^ RAGE expression increased following CNV induction, particularly in infiltrating macrophages and microglia, both of which are crucial to CNV development.^43^^,^^48^^,^^49^ Our findings further support the role of RAGE in subretinal fibrosis, as evidenced by increased expression of RAGE, particularly in the fibrotic area, as well as in the outer and inner plexiform layers and the ganglion cell layer (GCL). However, additional studies are needed to delineate the specific cell types in the outer retina that express RAGE during the progression of fibrosis.

Given the established role of RAGE in early neovascularization,^43^ we investigated its role in subretinal fibrosis associated with CNV. RAGE is expressed in various retinal cell types, including glial cells, Müller cells, neurons, vascular endothelial cells, pericytes, and RPE cells, with increased expression in pathological neovascular membranes and in RPE cells adjacent to drusen.^50^^–^^52^ Activation of the AGE–RAGE signaling pathway in these cells induces RPE activation, increases VEGF expression, and contributes to retinal endothelial damage and neovascularization.^53^^,^^54^

To consider the influence of age on the development of neovascular AMD,^55^^,^^56^ we used older mice, 15 to 17 months old. As seen in the CNV model with increased lesion size,^43^ fibrosis development was notably higher in WT mice, as indicated by multiple assays and analyses of fibrosis markers. These findings align with previous studies showing that RAGE activation exacerbates fibrosis in various tissues, including the lung, kidney, liver, and lens.^24^^,^^33^^,^^57^^,^^58^ In RAGE^−/−^ mice, the area and volume of fibrosis, along with Smad2 activation and fibrotic marker expression, were significantly reduced. Specifically, the absence of RAGE signaling led to decreased pSmad2 activation, accompanied by a lower expression of αSMA, which is a hallmark of myofibroblast activation, and type I collagen, a key extracellular matrix protein involved in fibrotic scar development. This reduction in fibrosis was correlated with lower levels of inflammation and oxidative stress, which have been linked to RAGE deficiency.^33^^,^^43^^,^^47^^,^^59^ Dysfunctional RPE cells secrete pro-inflammatory cytokines and chemokines and recruit innate immune cells, including microglia and macrophages.^60^ In subretinal fibrosis, infiltrating macrophages recruit choroidal fibroblasts and circulating fibrocytes and release profibrotic mediators that induce EMT in RPE cells or EndMT in choroidal vessels.^9^^,^^61^ Macrophages, particularly the M2 phenotype, are the most prominent inflammatory cells around neovascular lesions.^62^^–^^64^ RAGE is notably overexpressed in infiltrated M2 macrophages in fibrotic heart tissue, and its absence reduced M2 macrophage infiltration and fibrosis severity.^65^ Our data corroborated these findings, showing larger fibrotic lesions and significantly elevated levels of RAGE in WT mice with laser-induced subretinal fibrosis, accompanied by an increased presence of M2 macrophages. In contrast, RAGE^−/−^ exhibited reduced M2 macrophage infiltration and attenuated fibrosis.

Previous studies have also demonstrated significant RAGE upregulation within CNV lesions, accompanied by increased macrophage infiltration and elevated levels of profibrotic cytokines^43^ Consistent with these findings, we observed increased RAGE expression within fibrotic scar tissue, along with enhanced macrophage presence, further supporting the role of RAGE signaling in fibrosis. RAGE interacts with multiple ligands, including AGEs, HMGB1, and S100 proteins, to activate downstream pathways such as nuclear factor kappa B (NF-κB) and mitogen-activated protein kinase (MAPK), promoting transcription of pro-inflammatory and profibrotic genes. Notably, following CNV induction in WT mice, the pro-inflammatory ligand S100B is significantly upregulated and strongly localized at CNV sites, contributing to macrophage activation and tissue damage.^43^ Based on these results, we hypothesize that RAGE deficiency disrupts ligand–receptor interactions, leading to decreased macrophage infiltration, reduced expression of profibrotic mediators, and attenuation of ECM remodeling, thereby limiting fibrosis progression. Furthermore, RAGE deficiency has been associated with a shift in macrophage polarization from the pro-inflammatory M1 phenotype to the anti-inflammatory M2 phenotype, facilitating resolution of inflammation and reducing fibrogenesis.^66^ Similar effects have been observed in cardiac tissue, where RAGE deficiency reduces infiltration of M2-type profibrotic macrophages, resulting in diminished interstitial fibrosis and improved cardiac function.^65^ RAGE signaling also enhances vascular permeability and promotes leukocyte extravasation; thus, its inhibition may help maintain vascular integrity and restrict immune cell infiltration.^67^ Additionally, elevated antioxidant expression, such as superoxide dismutase 2 (SOD2), may contribute to the reduced fibrosis observed in RAGE^−/−^ mice.^47^ We hypothesize that a greater inflammation in aged WT mice, compared to RAGE^−/−^ mice, results from proinflammatory signaling cascades initiated by AGE–RAGE interactions, activating the NF-κB pathway ^68^^,^^69^ and subsequently promoting myofibroblast activation and ECM deposition. However, further investigation is needed to explore the role of RAGE in immune cell behavior, particularly macrophage polarization, and whether TGF-β induces RAGE expression in RPE cells.

EMT is a key mechanism in fibrosis development.^11^^,^^24^^,^^70^^,^^71^ To explore the role of RAGE in retinal fibrosis, we examined the effects of RAGE inhibition on EMT in hRPE cells. Treatment with RAGE antagonists, FPS-ZM1 and azeliragon, significantly inhibited TGF-β2–induced EMT without affecting cell viability, suggesting that the antifibrotic effects are not due to cytotoxicity. This supports the potential clinical application of RAGE inhibitors in retinal diseases without compromising cell survival. TGF-β2, a potent EMT inducer in retinal cells, is implicated in various fibrotic retinal diseases.^72^^,^^73^ Although not directly investigated in our study, previous reports have shown that TGF-β1 treatment increases RAGE expression in kidney proximal tubular cells,^74^ indicating a complex crosstalk mechanism between RAGE and TGF-β in disease progression. Inhibition of RAGE reduced mesenchymal markers, including αSMA, fibronectin, and collagen I, highlighting the role of RAGE in the TGF-β2–mediated fibrotic response in the retina. These findings are consistent with previous studies showing that RAGE promotes TGF-β–induced EMT and fibrosis in other tissues, such as the lens, liver, kidney, and lung.^24^^,^^33^^,^^74^

At the molecular level, RAGE-mediated fibrosis is known to involve several signaling pathways.^27^^,^^59^^,^^74^ In our study, we focused on the Smad2 pathway, a key mediator of TGF-β–induced fibrosis, based on previous findings.^24^^,^^70^ Our data demonstrated that TGF-β induced mitochondrial ROS production, which is consistent with previous findings^70^^,^^71^ and is required for TGF-β–mediated transcription of profibrotic genes.^75^ Mitochondrial ROS play a key role in enhancing TGF-β signaling through Smad2 phosphorylation.^70^^,^^76^ Smad2 signaling drives the transcription of fibrotic genes and promotes ECM production.^77^ Our data showed that TGF-β2 treatment significantly increased the phosphorylation of Smad2 in hRPE cells, an effect that was effectively inhibited by the RAGE antagonist FPS-ZM1. Supporting these findings, earlier studies showed that TGF-β levels increased significantly from 1 to 4 weeks after laser-induced CNV, accompanied by enhanced phosphorylation of Smad2 and Smad3.^78^^,^^79^ Inhibition of TGF-β signaling suppressed Smad2/3 phosphorylation and reduced the expression of angiogenic and inflammatory factors.^79^^,^^80^ Our in vivo data further confirmed this mechanism, showing increased RAGE expression and enhanced Smad2 phosphorylation in fibrotic lesions, a phenomenon that was markedly suppressed in RAGE^−/−^ mice. This finding suggests that RAGE facilitates TGF-β2–induced fibrotic signaling through Smad2 activation. No significant changes were observed in the phosphorylation of ERK, indicating that RAGE specifically modulates the canonical TGF-β/Smad signaling pathway in retinal cells. These findings are consistent with previous studies showing that RAGE activation enhances TGF-β signaling in multiple cell types.^24^^,^^70^^,^^81^

The mechanisms through which RAGE contributes to TGF-β2–mediated signaling are not completely understood. Evidence shows that RAGE inhibition attenuates mitochondrial oxidative stress and canonical TGF-β signaling,^82^ suggesting a mechanistic convergence that may amplify fibrogenic responses. Activation of RAGE by ligands (e.g., HMGB1, AGEs, S100) may initiate intracellular signaling cascades, including NF-κB and ERK1/2, which promote TGF-β2 transcription and increase TGF-β2 production. This ligand-induced signaling may establish a microenvironment that favors fibrosis by amplifying both inflammatory and fibrotic pathways. Supporting this, our data showed that RAGE expression was elevated in fibrosis alongside increased Smad2 phosphorylation, whereas genetic deletion of RAGE reversed these changes and significantly reduced fibrosis. Moreover, TGF-β itself can impair mitochondrial function and increase ROS generation,^83^ forming a positive feedback loop that exacerbates oxidative stress and fibrogenesis. Yet another possibility is that TGF- β2 signaling could promote the release of a RAGE ligand into the extracellular space, which in turn binds and activates RAGE, promoting the EMT of RPE cells. High mobility group box 1 (HMGB1) is likely a candidate ligand. However, further research is required to address these issues.

In conclusion, this study provides strong evidence that RAGE mediates the pathogenesis of subretinal fibrosis following retinal injury. The absence of RAGE resulted in reduced inflammation and fibrosis along with myofibroblast activation and type I collagen deposition. Additionally, RAGE inhibition with small-molecule antagonists FPS-ZM1 and azeliragon, effectively attenuated TGF-β2–induced EMT in hRPE cells, primarily through the inhibition of mitochondrial superoxide generation and suppression of Smad2 signaling. These findings highlight RAGE as a key target in retinal fibrosis and suggest that blocking RAGE could offer a therapeutic strategy for treating subretinal fibrosis in nAMD.

## Supplementary Material

Supplement 1
