# Supplementary material for: RAGE Is Essential for Subretinal Fibrosis in Laser-Induced Choroidal Neovascularization: Therapeutic Implications
Source: Invest Ophthalmol Vis Sci. 2025 Jun 9;66(6):30. doi: 10.1167/iovs.66.6.30 (PMC12161369; doi:10.1167/iovs.66.6.30)
Supplement: Supplement 1 [file iovs-66-6-30_s001.pdf]

Supplementary Figures

Supplementary Figure 1

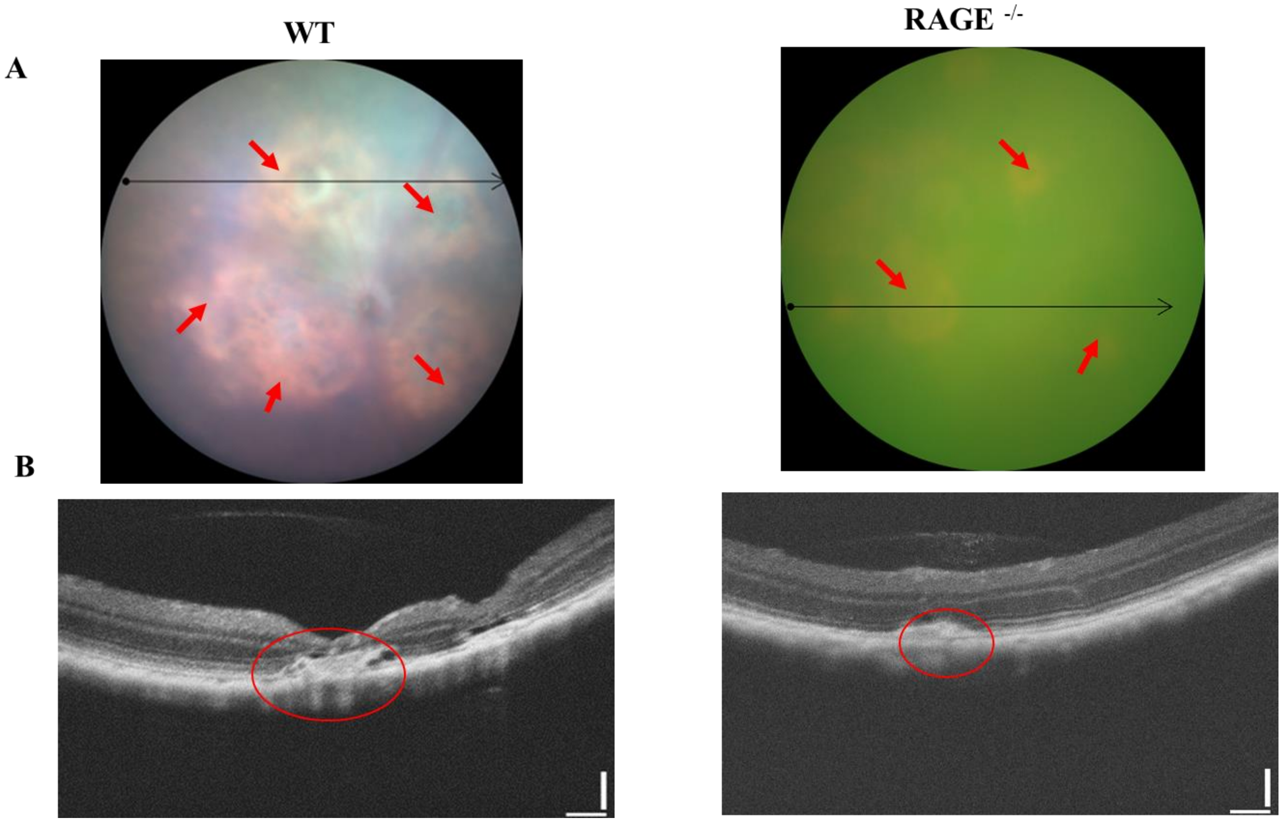

Supplementary Figure 1. RAGE $^{-/-}$ mice showed a reduction in subretinal fibrosis. Representative fundus images (**A**) and OCT (**B**) of subretinal lesions 35 days post-laser. Lesions in fundus (upper panel) were indicated with red arrows and subretinal fibrosis (lower panel) was indicated with red circles (n= 5-6). RAGE$^{-/-}$ mice carried a reporter green fluorescent protein, the expression of limited our ability to capture better fundus images. However, our technique resulted in the breakage of Bruch's membrane and scar formation in the subretinal space.

Supplementary Figure 2

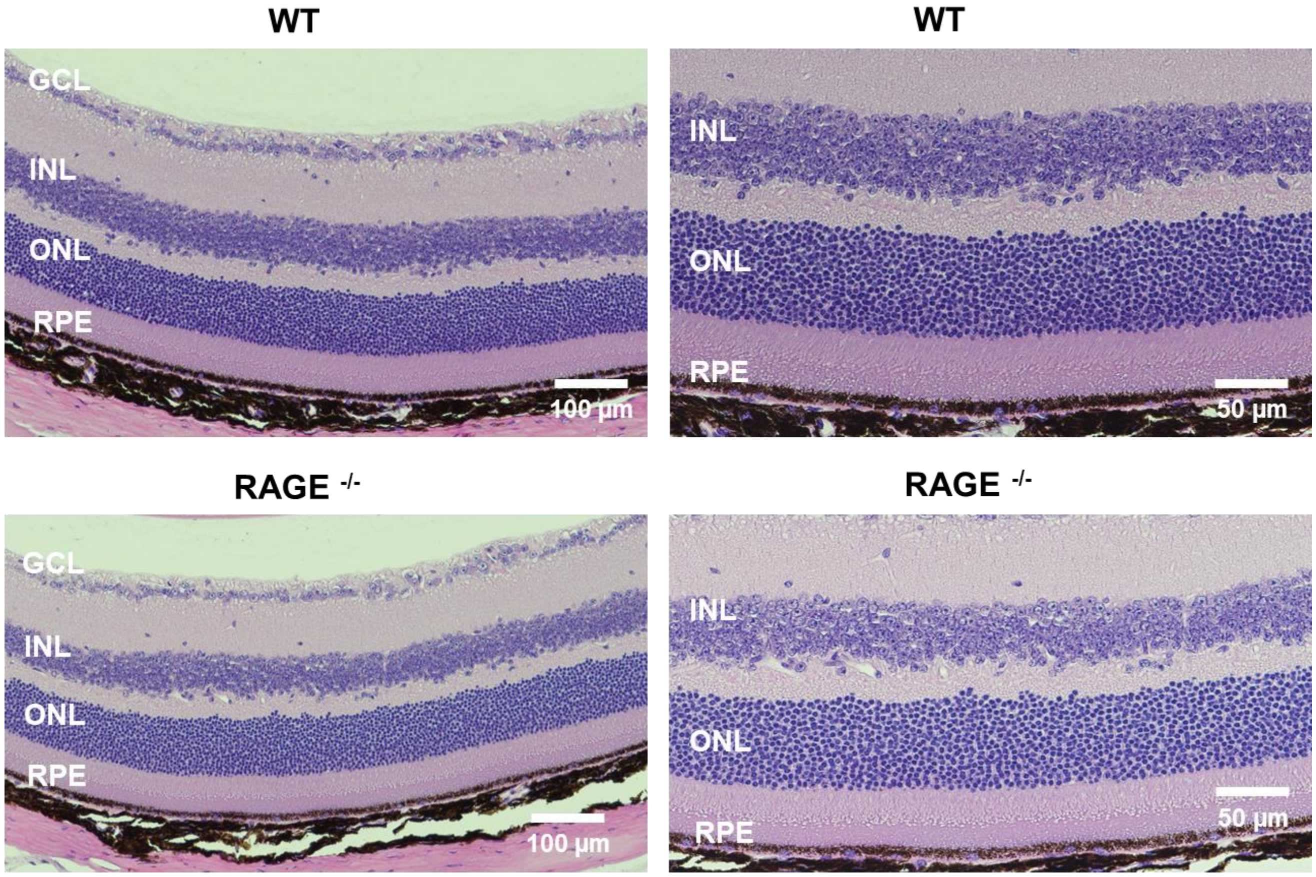

Supplementary Figure 2. Histological analysis of retinas from 26 week old WT and RAGE$^{-/-}$ mice. No apparent retinal phenotype was observed in RAGE$^{-/-}$ mice compared to WT controls. Low (left) and high (right) magnification images of the H&E-stained sections are shown (n = 4 sections from 2 mice).

Supplementary Figure 3

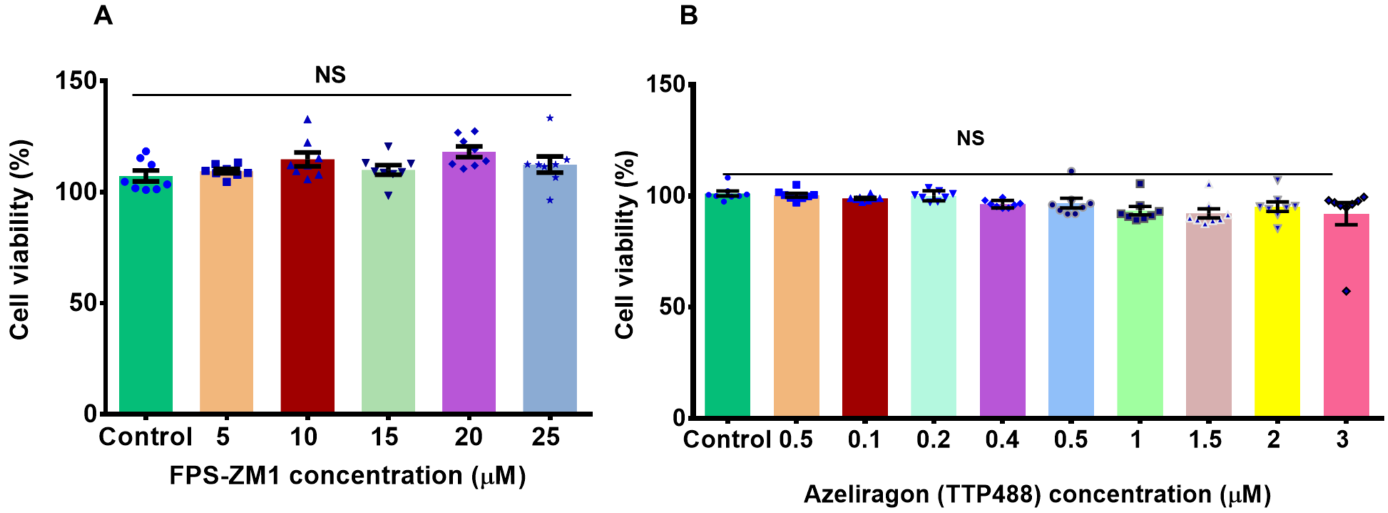

Supplementary Figure 3. Effect of RAGE antagonists (FPS-ZM1 or Azeliragon) on RPE Cell Viability: hRPE cells (25,000/well) were treated with varying concentrations of FPS-ZM1 (A) or Azeliragon (TTP488) (B) in DMEM containing 0.1% FBS for 48 hours. Cell viability was assessed by AlamarBlue assay. Data are presented as mean ± SEM (N=8). One-way ANOVA showed no significant differences ($p>0.05$). *NS: Not significant*.

Supplementary Figure 4

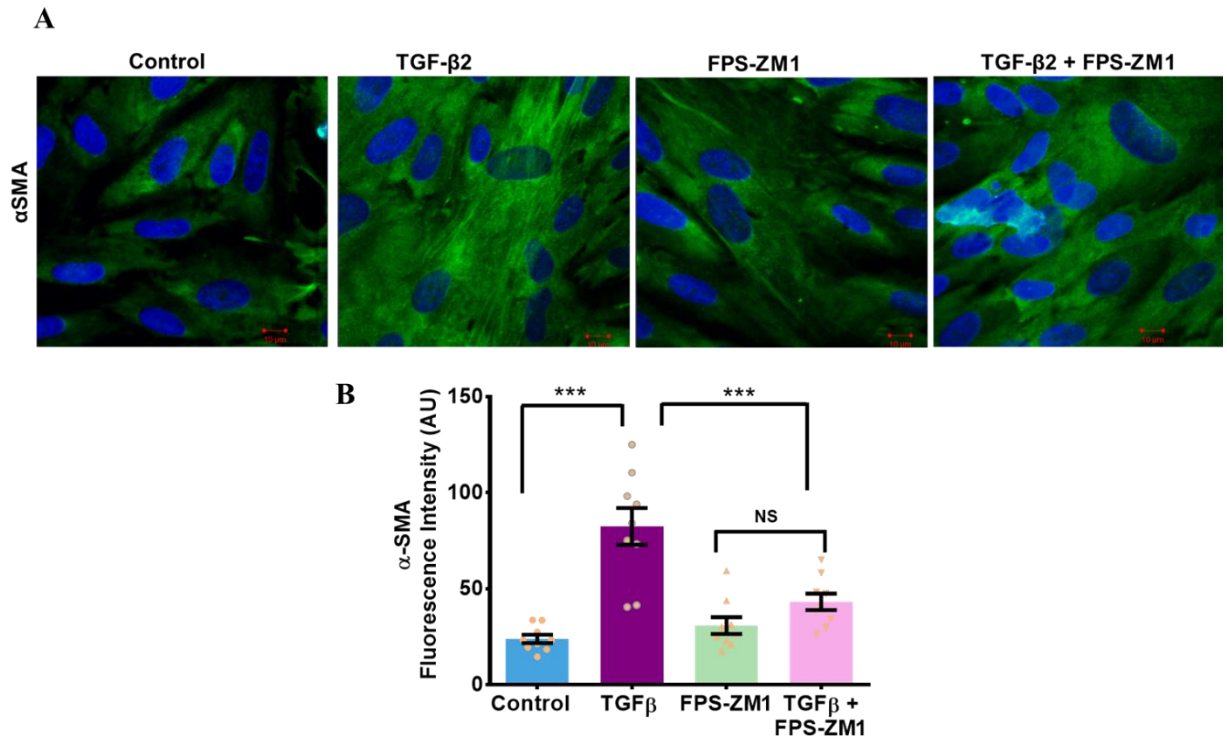

Supplementary Figure 4. RAGE antagonist inhibited α-SMA expression in hRPE cells. (**A**) hRPE cells were pretreated with FPS-ZM1 at 5 µM for 2 hours, followed by treatment with TGF-β2 (10 ng/mL) for an additional 48 hours in 4-well chamber slides containing medium with 0.1% FBS. Subsequently, cells were immunostained for α-SMA (green). The nuclei (blue) were stained with Hoescht. Scale bar: 10 µm. (**B**) Fluorescence intensity was quantified using Zen Software (Zeiss). Data are presented as mean ± SEM (N = 9, One-way ANOVA, Tukey-Kramer post-test, *** $p< 0.001$). NS: Not Significant.
